# Quantitative peri-lesional densitometry mapping via thin-slice volume rendering enhances differentiation of pneumonic-type lung cancer and inflammatory pneumonia

**DOI:** 10.3389/fonc.2026.1620815

**Published:** 2026-04-28

**Authors:** Sifan Chen, Ke Zhang, Min Zhao, Wentao Zhang, Li Tao, Qi Li, Fajin Lv

**Affiliations:** Department of Radiology, The First Affiliated Hospital of Chongqing Medical University, Chongqing, China

**Keywords:** computed tomography, densitometry, pneumonia, pneumonic - type lung cancer, thin-slice volume rendering

## Abstract

**Objectives:**

Pneumonic-type lung cancer (PTLC) poses significant diagnostic challenges owing to its overlapping computed tomography (CT) imaging appearance with inflammatory pneumonia. We developed and validated a novel diagnostic model that integrates densitometry-derived thresholds into modified thin-slab volume rendering (tsVR) to differentiate PTLC from inflammatory pneumonia. We further evaluated and compared the diagnostic performance of this tsVR approach against conventional CT feature-based diagnosis.

**Materials and methods:**

This retrospective study enrolled 383 patients (193 PTLC, 190 pneumonia) for model development (training/internal validation cohorts, 7:3 ratio) and 61 patients as external validation cohort. Peri-lesional densitometric analysis identified voxel-level and individual-level CT density thresholds translated into modified tsVR parameters to enhance the peri-lesional visualization. Discriminating capabilities of tsVR were assessed by four radiologists against original CT feature-based diagnosis using the area under curve (AUC), sensitivity, specificity, accuracy, positive predictive value (PPV), negative predictive value (NPV), F1-score, F2-score and Matthews correlation coefficient (MCC), followed by logistical regression analyses assessing independent influencing factors for diagnostic accuracy.

**Results:**

The tsVR model achieved superior performance over original CT feature-based diagnosis across all metrics, with internal validation AUC 0.86 (sensitivity 0.90, specificity 0.82, accuracy 0.86, F2 score 0.89) and external validation AUC 0.81 (sensitivity 0.90, specificity 0.73, accuracy 0.82, F2 score 0.87). Logistical regression analyses confirmed robustness of tsVR in diagnostic accuracy across scanners, scanning doses and reconstruction protocols, with good inter-observer agreement (κ=0.713).

**Conclusion:**

The tsVR significantly improves the diagnostic efficacy in discriminating PTLC from inflammatory pneumonia, demonstrating clinical applicability and robust stability.

## 1 Introduction

Lung cancer remains the leading cause of cancer-related mortality worldwide. CT screening effectively reduces mortality via early detection of lung cancer (1). However, it remains challenging for radiologists to diagnose pneumonic-type lung cancer (PTLC), a subtype manifesting as nonobstructive focal or diffuse pulmonary consolidation mimicking inflammatory pneumonia on CT (2, 3). Historically, radiologists have relied on conventional CT morphological features to aid in differentiation, such as lobular fissure bulging, air bronchogram distortion, margin characteristics, necrosis, pleural attachment, and the CT angiogram sign. However, owing to overlapping imaging manifestations and the limited ability to reliably discern discriminative CT features, nearly 40% of PTLC patients were initially misdiagnosed as pneumonia (4), which often resulted in delayed management and poorer prognosis (5).

Radiomics emerges as a transformative paradigm, enabling the extraction of subvisual quantitative biomarkers from medical images (6). Notably, peri-lesional heterogeneity reflecting tumor-stroma interactions and microenvironment remodeling has demonstrated diagnostic value in lung cancer classification and prediction (7). Nevertheless, clinical translation of radiomic models remains constrained by technical barriers such as feature instability across reconstruction protocols, poor interpretability of high-dimensional classifiers, and workflow incompatibility with routine diagnostic pipelines (8, 9). These challenges necessitate the development of hybrid solutions integrating computational biomarkers with clinically practical diagnostic tools.

Thin-slice volume rendering (tsVR), a well-established postprocessing technique, enables selective tissue visualization through density-dependent pseudocolor mapping and opacity modulation (10, 11). We hypothesize that the optimized tsVR technique could enhance the visualization of the peri-lesional heterogeneity so that to address the PTLC-pneumonia diagnostic dilemma. Our approach involves: identifying density thresholds from peri-lesional densitometric patterns to distinguish malignant consolidations and translating the thresholds into tsVR parameters to enhance the visualization of diagnostically critical densitometric differences.

This study aims to develop and validate a modified tsVR diagnostic system integrating densitometric thresholds, evaluate its impact on diagnostic performance in comparison with the original CT-feature based diagnosis, and analyze potential factors influencing its clinical implementation.

## 2 Methods

### 2.1 Patients

**Figure 1:**
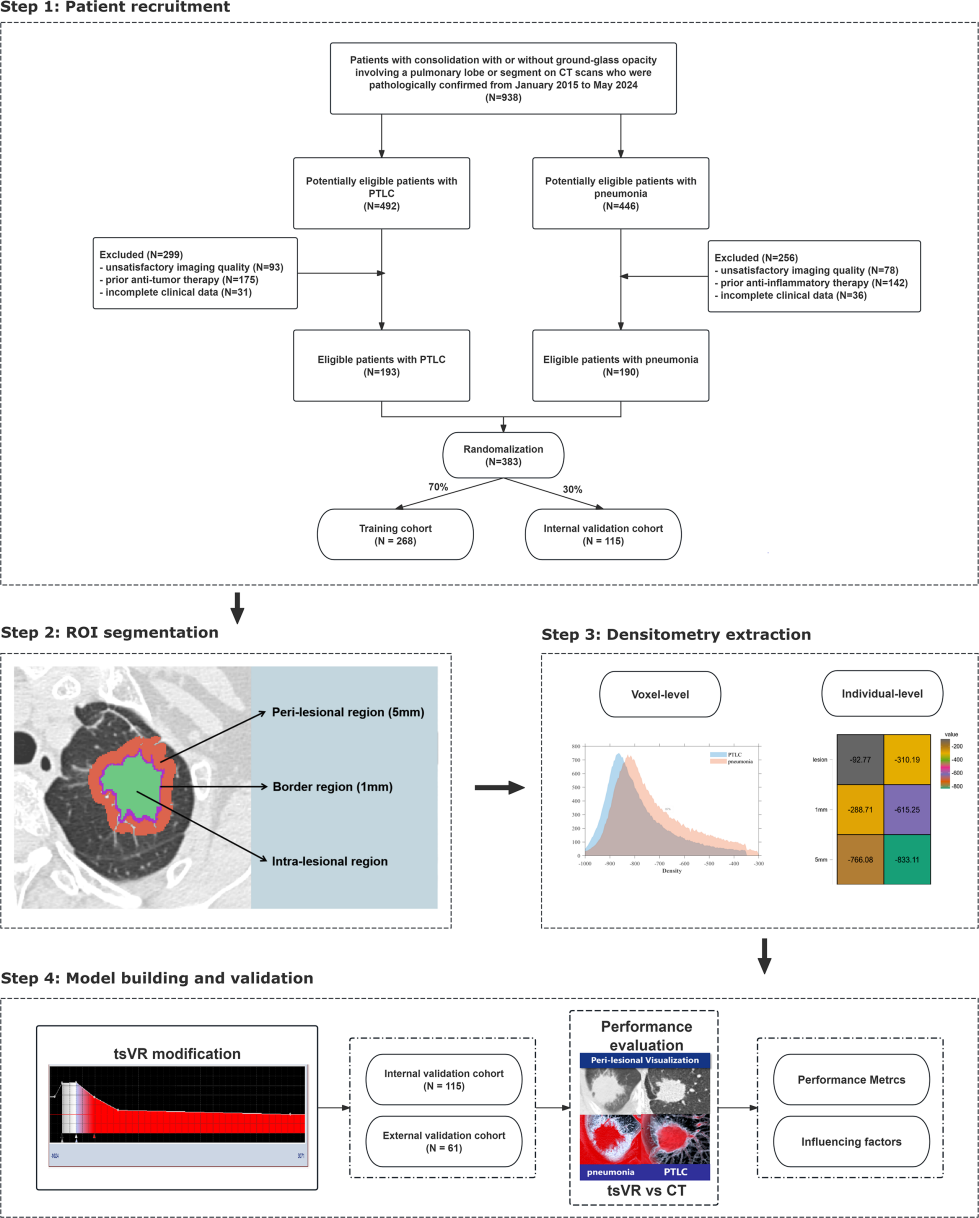
Flowchart shows patient recruitment and overall experimental design for this study. PTLC, pneumonic-type lung cancer, tsVR, thin-slice volume rendering.

This retrospective study, conducted in compliance with the Declaration of Helsinki, was approved by the ethics committee of the First Affiliated Hospital of Chongqing Medical University (K2024-057-01), with a waiver of informed consent, as de-identified data were utilized and no protected health information (PHI) was required. The primary cohort included patients from January 2015 to May 2024 meeting these criteria: (1) CT scanning indicated consolidation with or without ground-glass opacity involving a pulmonary lobe or segment; (2) patients underwent noncontrast chest CT at our institute within 2 weeks before pathologic evaluation; (3) patients with a pathological diagnosis confirmed via surgical resection, transbronchial lung biopsy, or percutaneous lung biopsy. Meanwhile, the exclusion criteria were as follows: (1) patients with unsatisfactory imaging quality due to respiratory motion artifact; (2) patients with prior anti-tumor or anti-inflammatory therapy before initial chest CT scanning; (3) patients with incomplete clinical data. Finally, a total of 383 patients (193 PTLC and 190 inflammatory pneumonia) from our institute were consecutively enrolled to minimize selection bias and randomized with a ratio of 7:3 into the training cohort (268) and the internal validation cohort (115). (**Figure 1**) To evaluate model generalizability, we established an independent external validation cohort comprising consecutively enrolled patients (n=61, 31 PTLC and 30 inflammatory pneumonia) from a geographically distinct branch hospital. This facility utilizes separate patient registration systems and imaging acquisition protocols but maintains diagnostic standards equivalent to those of the primary hospital. All inclusion/exclusion criteria were strictly preserved during case selection within the same timespan to ensure methodological consistency. The detailed patient characteristics in the training and validation cohorts were summarized in **Supplementary Table 1**.

### 2.2 CT protocols

Unenhanced chest CT scans were performed using the multidetector CT scanners (Somatom Force, Siemens Healthcare; Somatom Definition Flash, Siemens Healthcare; Somatom Perspective, Siemens Healthcare; Discovery 750, GE Healthcare, etc.). All patients underwent CT scan in a supine position at the end of inspiration during a single breath hold to prevent respiratory motion artifacts. The CT scanning parameters were as follows: tube voltage, 100–130 kVp; tube current, 100–250 mA; slice thickness, 5.0 mm and slice interval, 5.0 mm. All images were reconstructed at 1.0mm section thickness with 1.0 mm slice interval.

### 2.3 Lesion segmentation and densitometry extraction

The regions of interest (ROI) were manually segmented by two thoracic radiologists blinded to clinical information (with 6 and 12 years of experience) using an open-source software (3D-Slicer^®^, version 5.6.2). The lesions were segmented layer-by-layer using a freehand tool to annotate the outer boundary of the lesion. Three types of ROIs, including the lesion ROI, peri-lesional 5-mm ROI and boundary 1-mm ROI were annotated from the CT images. The peri-lesional and boundary compartment around the lesion was defined via the use of morphological operations dilation and shrink as a region expansion or erosion from the lesion boundary to 5 mm and 1mm, respectively. The choice of peri-lesional compartment size was determined based on the previous studies where the most discriminating features were found to be within an immediate distance of 5 mm from the lesion (12, 13). A 1-mm boundary distance was selected to differentiate the density variations between the peri-lesional area and the lesion. Pleural wall and vessels were avoided and removed in the process of obtaining ROI. Subsequently, the CT density values of all voxels within these ROIs were extracted using a custom MATLAB script (R2018b; The MathWorks, Inc., Natick, MA, USA) (**Supplementary Material**).

### 2.4 Development of tsVR model and image analysis

Thin-slab Volume rendering (tsVR) is a three-dimensional post-processing technique that assigns continuous opacity values and colors to each voxel based on Hounsfield units (HU), enabling visualization of tissue gradients and spatial relationships. To transform quantitative peri-lesional density variations identified through densitometry into an interpretable imaging biomarker, we developed customized parameter for tsVR to effectively amplify density heterogeneity within peri-lesional areas. This enhanced peri-lesional imaging pattern, visualized as a “halo” sign on tsVR reconstructions, served as the key discriminator for differentiating inflammatory pneumonia from PTLC. Four radiologists (K.Z., S.F.C, W.T.Z and F.J.L with 3, 5, 7 and 20 years of experience in chest imaging) independently reviewed tsVR images from both internal and external test set on a PACS workstation (Carestream Vue, version 12.2.6.3000020). For tsVR post-processing, all CT images have been properly imported to the PACS clients, where the software allows real-time 3D processing of tsVR reconstruction without manual user interaction. Each observer was permitted exclusively to perform independent diagnostic assessments on tsVR images, with deliberate exclusion of access to corresponding original CT images to minimize potential bias. The opacity-CT value curve was preset in the PACS client and was provided in **Supplementary Figure 1**. Fixed tsVR parameters were employed for image reading, with no manual adjustments permitted, to ensure strict technical reproducibility and the elimination of observer and measurement biases. Prior to the formal image analysis, all observers had received training in PACS operation and were familiar with tsVR images. Another independent radiologist (M.Z), not involved in the tsVR assessment, recorded the original diagnosis and all related technical information including scanner, radiation dose, reconstruction kernel and algorithm. The original diagnosis was based on traditional CT morphological features (e.g., margin characteristics, air bronchogram pattern, interlobular fissure bulging) and established through consensus review by at least three radiologists.

### 2.5 Statistical analysis

All analyses were performed using software SPSS (version 26.0; SPSS, Chicago, IL) and statistical program R (version 4.4.1, https://www.r-project.org/). The diagnostic performance of the tsVR-based model was comprehensively evaluated using established classification metrics, including the area under curve (AUC), sensitivity, specificity, accuracy, positive predictive value (PPV), negative predictive value (NPV), F1-score, F2-score (prioritizing sensitivity) and Matthews correlation coefficient (MCC). The detailed formula for these metrics were provided in **Supplementary Materials**. Interobserver agreement was assessed using kappa statistics. The Chi-square test and Mann–Whitney U test were used to assess the statistical difference of the baseline characteristics. To investigate factors influencing the diagnostic accuracy of the models in differentiating PTLC from pneumonia, univariate logistical regression was performed to assess the association addressing clinical and technical influencing factors: clinical factors - pathological diagnosis (lung cancer/pneumonia), histological subtype and tumor grade (high/moderate/low); technical factors - scanner (Siemens/GE/others), radiation dose (low: ≤2 mSv; standard: >2 mSv), reconstruction kernel (sharp/medium/smooth), and algorithm type (iterative reconstruction [IR]/filtered back-projection [FBP]). Statistical significance was defined as two-tailed *P* <.05.

## 3 Results

### 3.1 Patient characteristics

The clinical data and baseline characteristics of the patients in the training and validation cohorts are summarized in **Supplementary Table 1**. In the training cohort, the median age (interquartile range, IQR) was 62.00 (53.00–68.50) years for patients with PTLC and 56.00 (50.00–66.00) years for patients with pneumonia. Patients with PTLC were significantly older than those with inflammatory pneumonia (*P* = 0.002). Similarly, in the internal validation cohort, the median age (IQR) was 65.50 (57.25–68.00) years for PTLC and 54.00 (48.00–67.00) years for pneumonia (*P* = 0.003). In the external validation cohort, the median ages were 66.00 (53.50–68.00) years and 64.50 (56.50–70.50) years for the PTLC and pneumonia groups, respectively, with no significant difference between the two groups (*P* = 0.549). Regarding the specific pathological diagnoses, among the 135 patients with PTLC in the training cohort, 113 (83.70%) were confirmed with invasive adenocarcinoma, 5 (3.70%) with invasive mucinous adenocarcinoma, and 17 (12.59%) with other malignant subtypes. For the 133 patients with inflammatory pneumonia in the training cohort, the pathological diagnoses included 87 (65.41%) with non-specific pneumonia, 30 (22.56%) with tuberculosis, and 16 (12.03%) with inflammatory pseudotumors. The distribution proportions of these pathological subtypes were consistent across the internal validation (n=115) and external validation (n=61) cohorts, ensuring a well-balanced dataset for subsequent model evaluation.

### 3.2 Quantitative analysis of peri-lesional and boundary regions

**Figure 2:**
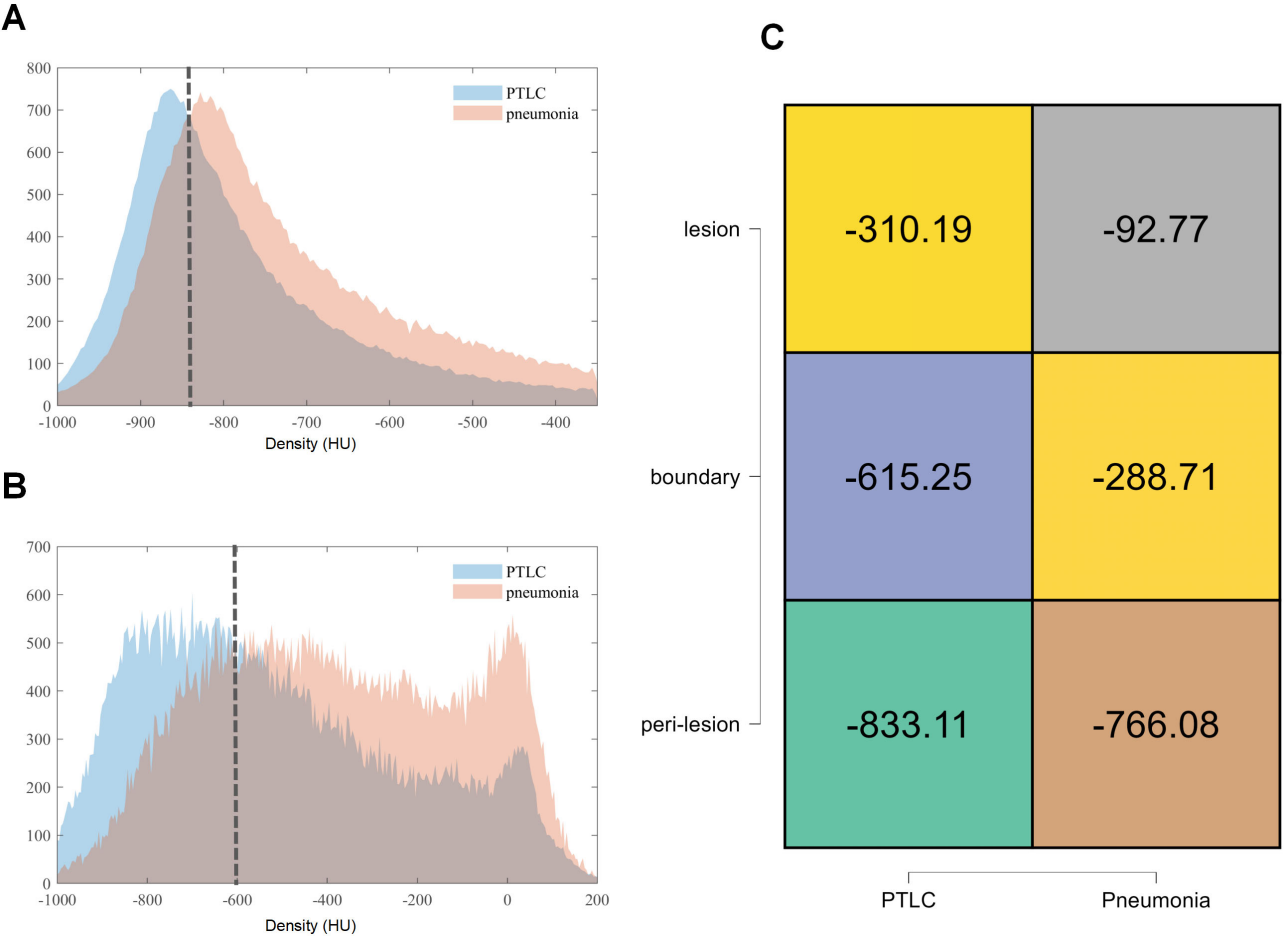
Densitometric differences of pneumonic-type lung cancer and pneumonia at voxel **(A, B)** and individual **(C)** levels within the 5 mm peri-lesional **(A, C)** and 1 mm boundary **(B, C)** regions.

**Figure 2** illustrates distinct CT density distributions in PTLC compared to pneumonia, analyzed at both individual and voxel levels within peri-lesional (5-mm) and boundary (1-mm) regions. At the individual level, pneumonia exhibited a significantly higher median CT value (-766 HU) in the 5-mm peritumoral region compared to PTLC (-833 HU) (*P* < 0.05) (**Figure 2C**). To derive the precise diagnostic thresholds, voxel-level histogram analysis was performed, which identified an optimal density threshold of -830 HU to differentiate pneumonia from PTLC.(**Figure 2A**) In the 1 mm boundary region, PTLC exhibited a median CT value of -615 HU compared to -288 HU for pneumonia(**Figure 2C**), yielding a derived voxel-level cutoff at -600 HU (**Figure 2B**). To enhance visualization of peri-lesional regions, tsVR parameters were optimized to assign 70% opacity and white pseudocolor to voxels within the discriminative density range (-830 HU to -600 HU), which corresponded to the differential interval between PTLC and pneumonia (**Supplementary Figure 1**).

### 3.3 Diagnostic performance of tsVR

**Figure 3:**
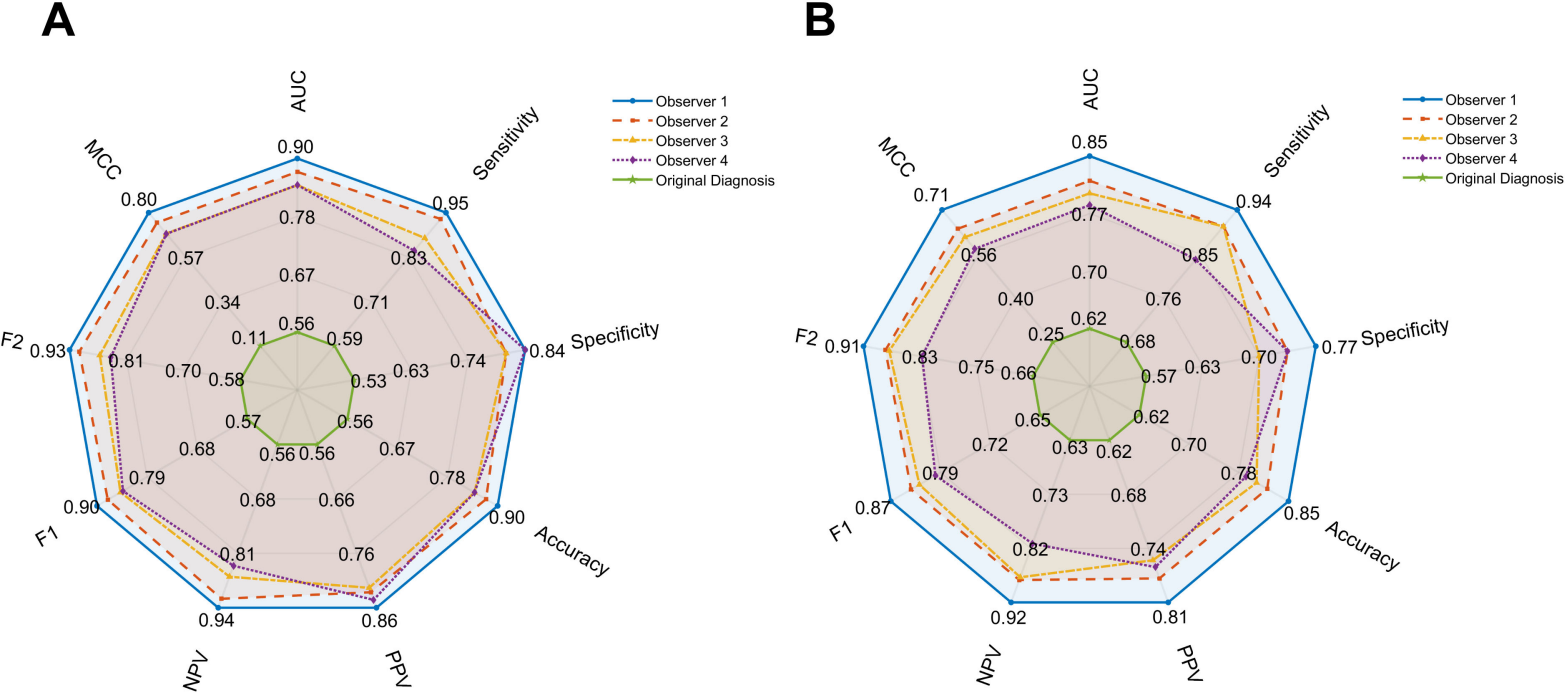
Radar plots show the diagnostic performance of tsVR (four observers) and CT across ten metrics in internal validation set **(A)** and external validation set **(B)**. AUC, the area under curve, PPV, positive predictive value, NPV, negative predictive value, MCC, Matthews correlation coefficient.

**Figure 4:**
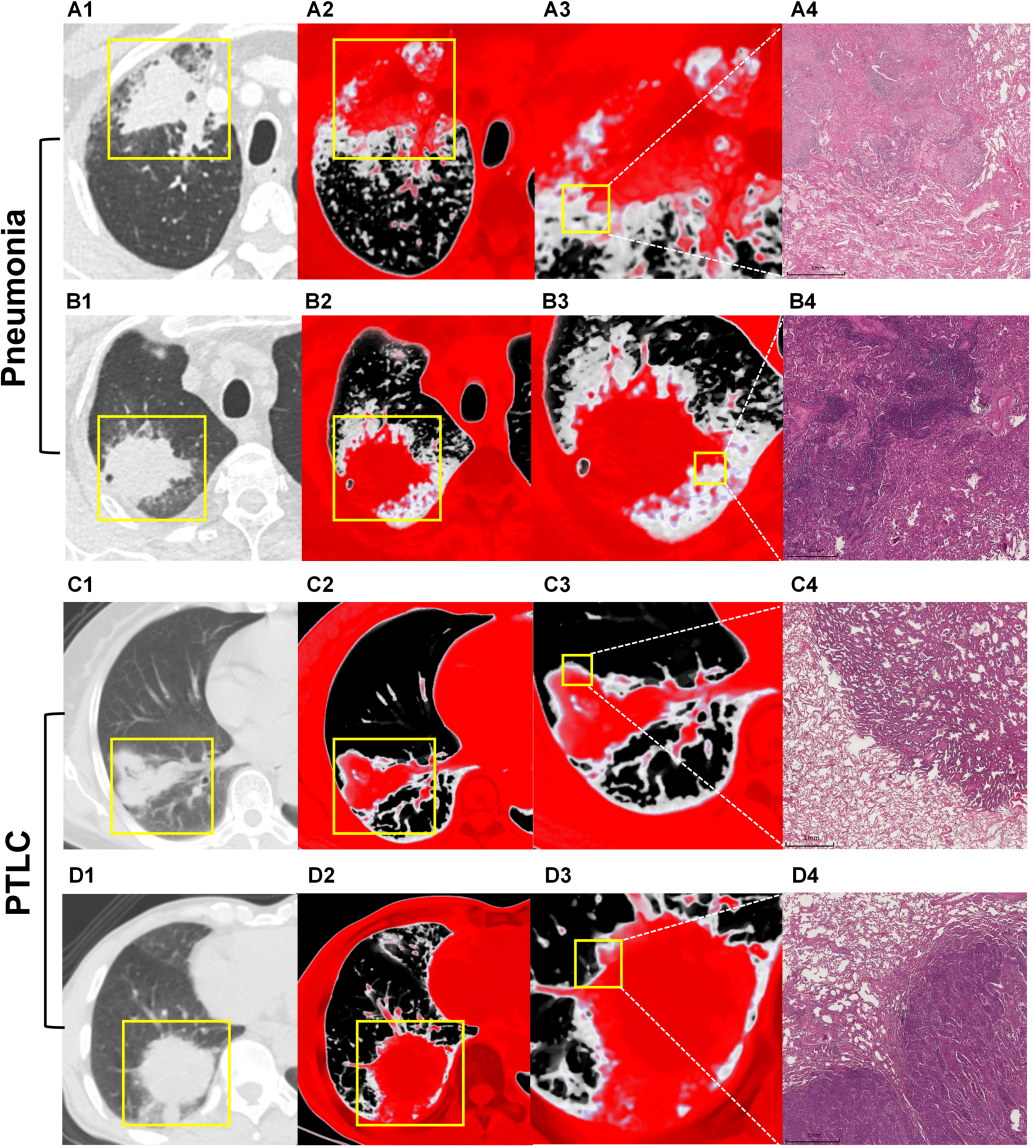
Images show representative unenhanced CT and tsVR images of pneumonia **(A, B)** and pneumonic-type lung cancer **(C, D)**. **(A)** A 60-year-old woman with Granulomatous Pneumonia. tsVR imaging **(A2, A3)** demonstrates enhanced delineation of peri-lesional increased density compared to conventional CT **(A1)**. Corresponding hematoxylin and eosin (H&E 2×) staining **(A4)** reveals an ill-defined histopathological boundary with reduced alveolar spaces in peri-lesional parenchyma; **(B)** A 61-year-old man with non-specific pneumonia. tsVR highlights diffuse ground-glass opacity surrounding the consolidation **(B2, B3)**, which is less conspicuous on CT **(B1)**. Histopathological analysis (B4) confirms inflammatory cell infiltration and indistinct lesion margins (4×). **(C)** A 79-year-old man with invasive adenocarcinoma. The well-defined lesion shows no ground-glass opacity on CT **(C1)** and tsVR indicate the absence of increased density in peri-lesional regions **(C2, C3)**. H&E (2×) staining **(C4)** shows a clear tumor-lung interface with preserved alveolar architecture in surrounding tissue, exhibiting larger alveolar spaces compared to inflammatory lesions. **(D)** A 71-year-old man with squamous cell carcinoma. tsVR corroborates the absence of peri-lesional opacity **(D2,D3)** compared with CT **(D1)**. Histopathology **(D4)** further confirms well-defined tumor borders(2×). PTLC = pneumonic-type lung cancer.

As summarized in **Table 1**, the tsVR-based diagnostic model outperformed the original CT feature-based diagnosis across all metrics in both internal and external validation cohorts. In the internal validation set, the tsVR model achieved an AUC of 0.86 (sensitivity: 0.90, specificity: 0.82, accuracy: 0.86, PPV: 0.84, NPV: 0.89, F2 score: 0.89), whereas the original CT feature-based model yielded an AUC of 0.56 (sensitivity: 0.59, specificity: 0.53, accuracy: 0.56, PPV: 0.56, NPV: 0.56, F2 score: 0.58). Similarly, in the external validation set, the tsVR model maintained robust performance with an AUC of 0.81 (sensitivity: 0.90, specificity: 0.73, accuracy: 0.82, PPV: 0.78, NPV: 0.87, F2 score: 0.87), compared to the original diagnosis yielding an AUC of 0.62 (sensitivity: 0.68, specificity: 0.57, accuracy: 0.62, PPV: 0.62, NPV: 0.63, F2 score: 0.66). **Figure 3** demonstrates the superior diagnostic performance of the tsVR model across all four radiologists via radar plots, with substantial inter-observer agreement (Cohen’s κ = 0.713). Representative CT and tsVR images highlighting differential peri-lesional features are shown in **Figure 4**.

**Table 1:** Diagnostic performance.

Dataset	Image	AUC	Sensitivity	Specificity	Accuracy	PPV	NPV	F1	F2	MCC
Internal	tsVR	0.86	0.90	0.82	0.86	0.84	0.89	0.87	0.89	0.73
	CT	0.56	0.59	0.53	0.56	0.56	0.56	0.57	0.58	0.11
External	tsVR	0.81	0.90	0.73	0.82	0.78	0.87	0.83	0.87	0.64
	CT	0.62	0.68	0.57	0.62	0.62	0.63	0.65	0.66	0.25

AUC, the area under curve, PPV, positive predictive value, NPV, negative predictive value, MCC, Matthews correlation coefficient.

### 3.4 Analysis of influencing factors

**Table 2** indicated that pathological diagnosis (PTLC, OR = 2.28, 95%CI: 1.48 - 3.51, *P* < 0.001) and histologic subtype (Non-specific pneumonia, OR = 0.54, 95%CI: 0.32 - 0.91, *P* = 0.021; Tuberculosis, OR = 0.17, 95%CI: 0.09 - 0.31, *P* < 0.001) significantly influenced the diagnostic accuracy of tsVR. However, tumor grade and technical variables (e.g., equipment, scanning dose, reconstruction kernel, algorithm) exhibited no statistically significant influence on diagnostic accuracy (*P* > 0.05 for all). In contrast, the diagnostic accuracy of original CT feature-based diagnosis was significantly influenced by reconstruction kernel (smooth, OR = 0.40, 95%CI: 0.21 - 0.77, *P* = 0.006) and algorithm (FBP, OR = 0.39, 95%CI: 0.19 - 0.80, *P* = 0.010). (**Supplementary Table 2**) These findings underscore the stability and reproducibility of the tsVR model across diverse clinical and imaging conditions.

**Table 2:** Influencing factors associated with diagnostic accuracy for tsVR.

Variables	β	S.E	Z	*P*	OR (95%CI)
Histopathology					
Pneumonia					1.00 (Reference)
PTLC	0.82	0.22	3.74	**<.001**	2.28 (1.48 ~ 3.51)
Histological subtype					
Invasive adenocarcinoma					1.00 (Reference)
Invasive mucinous adenocarcinoma	-0.48	0.58	-0.84	0.402	0.62 (0.20 ~ 1.91)
Other	-0.48	0.58	-0.84	0.402	0.62 (0.20 ~ 1.91)
Non-specific pneumonia	-0.62	0.27	-2.31	**0.021**	0.54 (0.32 ~ 0.91)
Tuberculosis	-1.76	0.3	-5.79	**<.001**	0.17 (0.09 ~ 0.31)
Inflammatory pseudotumor	-0.22	0.51	-0.43	0.67	0.80 (0.29 ~ 2.20)
Tumor grade					
Low					1.00 (Reference)
Moderate	0.79	0.68	1.17	0.243	2.21 (0.58 ~ 8.33)
High	-0.08	0.72	-0.11	0.91	0.92 (0.22 ~ 3.81)
Equipment					
Siemens					1.00 (Reference)
GE	-0.15	0.21	-0.7	0.482	0.86 (0.56 ~ 1.31)
Other	1.4	0.74	1.89	0.059	4.04 (0.95 ~ 17.21)
Scanning Dose					
Standard (>2 mSv)					1.00 (Reference)
Low (≤2 mSv)	-0.01	0.26	-0.03	0.976	0.99 (0.60 ~ 1.65)
Reconstruction Kernel					
Smooth					1.00 (Reference)
Medium	-0.12	0.22	-0.55	0.586	0.89 (0.58 ~ 1.36)
Sharp	1.69	1.03	1.64	0.101	5.41 (0.72 ~ 40.63)
Reconstruction Algorithm					
FBP					1.00 (Reference)
IR	0.31	0.24	1.29	0.198	1.37 (0.85 ~ 2.20)

OR, Odds Ratio, CI, Confidence Interval, PTLC, pneumonic-type lung cancer, FBP, filtered back projection, IR, iterative reconstruction.Bold text indicates statistical significance.

## 4 Discussion

Pneumonia-type lung cancer (PTLC) represents a diagnostically challenging subset of lung malignancies, characterized by imaging features overlapping with pneumonia yet harboring starkly divergent prognoses. The absence of robust diagnostic methods renders PTLC highly susceptible to misdiagnosis, leading to substantial dependence on subjective radiologist expertise and resulting in delayed interventions that profoundly impact patient survival. To address this major diagnostic challenge, this study developed a novel diagnostic model based on tsVR by leveraging densitometry differences between pneumonia-type lung cancer (PTLC) and pneumonia. The tsVR-based diagnostic model demonstrated robust diagnostic performance (AUC: 0.86 internal, 0.81 external) and diagnostic accuracy (0.86 internal, 0.82 external), significantly outperforming original CT feature-based approaches. Beyond diagnostic accuracy, the model substantially reduced dependence on radiologist experience: four radiologists with varying expertise consistently achieved superior performance across all metrics using tsVR compared to traditional CT interpretation frameworks, alongside good inter-observer agreement (κ= 0.713), supporting a critical foundation for clinical utility. Notably, the tsVR model exhibited no significant variations in accuracy across imaging parameters, underscoring its superior stability and reproducibility relative to radiomic models. These findings collectively position tsVR as an efficient tool expected to be integrated in real-world clinical workflows for PTLC diagnosis while mitigating risks of unnecessary biopsies or delayed treatments.

Current literature lacks consensus on definitive CT features for distinguishing PTLC. (**Supplementary Table 3**) Previous studies have suggested potential CT features including lobular fissure bulging, air bronchogram distortion, necrosis, pleural attachment, satellite lesions, and the CT angiogram sign, but these findings were inconsistent across studies (14–16). This ambiguity has rendered clinical practice heavily reliant on radiologist experience, a subjective paradigm fraught with high misdiagnosis rates. In our cohort, 46% (40/87) of pneumonia patients were erroneously diagnosed with malignancy on initial CT, while 38% (34/89) of PTLC cases were misdiagnosed as pneumonia, with poor discriminative performance (AUC: 0.56–0.62, MCC 0.11-0.25). Such misdiagnosis frequently precipitates inappropriate management, including delayed interventions or unnecessary invasive procedures. In contrast, the tsVR model, which enhances peri-lesional visualization, demonstrated transformative diagnostic accuracy, achieving AUCs of 0.86 (internal) and 0.82 (external)—surpassing conventional CT-based approaches by >50% and 30%, respectively. Critically, tsVR’s superiority extended to metrics pivotal for clinical decision-making: it exhibited markedly higher PPV (0.84 vs. 0.56 internally; 0.78 vs. 0.62 externally), F2-score (0.89 vs. 0.58; 0.87 vs. 0.66), and model integrity (MCC: 0.73 vs. 0.11; 0.64 vs. 0.25), reflecting its robust ability to prioritize sensitivity in avoiding false-negative PTLC diagnoses. These advancements suggest tsVR’s potential to standardize diagnostic workflows, mitigate operator-dependent variability, and reduce the risks of delayed or invasive mismanagement.

While radiomics has emerged as a promising alternative by decoding tumor pathophysiology through quantitative image analysis, its clinical translation faces inherent barriers including technical complexity, workflow incompatibility, and vulnerability to imaging protocol variations. While combining radiomics with clinical data improves diagnostic accuracy (AUC: 0.85–0.89), these hybrid models still depend heavily on specialized expertise and computational pipelines that hindered clinical implementation (4, 15). Critically, radiomic analysis cannot be readily integrated into routine diagnostic workflows, as it typically requires dedicated analysis pipelines separate from clinical practice (17, 18). While some commercial computer-aided diagnosis (CAD) systems now offer automated radiomic feature extraction, significant challenges persist in establishing robust diagnostic support, such as identifying radiomic features that specifically differentiate PTLC from pneumonia and determining how to effectively incorporate such features into diagnostic models. In contrast, post-processing techniques like tsVR are inherently compatible with clinical workflows, having been pre-integrated into PACS software and routinely utilized for years. Previous studies demonstrated that tsVR enhances pulmonary lesion visualization through optimized opacity-CT value curve settings, significantly improving nodule detection rates and achieving 80.28% consistency with pathological measurements of invasive components (10, 11). Considering these advantages, we developed the tsVR model using densitometry, a simpler approach that has been extensively utilized in the diagnosis of diverse pulmonary pathologies including emphysema, air trapping, fibrosis, and infiltrative conditions (19, 20) and shows less variations by technical settings (9, 21). Unlike conventional radiomic features (e.g., texture and higher-order features) derived through complex mathematical transformations, which are often sensitive to image acquisition variations, the tsVR model leverages first-order features, which demonstrate high repeatability and reproducibility across variations in imaging acquisition parameters (22, 23). In the current study, the diagnostic accuracy of the tsVR model showed no significant difference across imaging parameters including slice thickness, reconstruction kernels, and radiation dose. These factors are well-established as major contributors to radiomic feature instability (24). Furthermore, the tsVR model maintained robust diagnostic accuracy when evaluated against other potential factors, such as scanner manufacturers and reconstruction algorithms, underscoring its technical generalizability.

Prior studies have demonstrated the diagnostic value of peri-tumoral radiomic features within 5 mm of lesions for distinguishing lung cancer from pneumonia, with peri-tumoral characteristics exhibiting superior stability compared to intralesional features (7, 9, 25). The distinct CT densitometric differences observed between PTLC and pneumonia in this study likely reflect underlying histopathological disparities. Previous studies suggested that the regional CT densities had significant linear correlation with the proportion of air in alveoli, so that the discriminative threshold of -830 HU to -600 HU indicate the abnormal replacement of healthy lung tissue (-1000 HU) with pathological components (26, 27). This fundamental reliance on absolute CT values underscores our rationale for exclusively utilizing unenhanced CT scans. While contrast-enhanced CT is valuable for evaluating tumor vascularity and characterizing pneumonia-like tumors, significant morphological and enhancement pattern overlaps often persist between PTLC and inflammatory pneumonia on enhanced images (15, 16). Furthermore, the intravenous administration of contrast agents would introduce substantial variability in CT density measurements due to differences in contrast phases and local hemodynamics. Such fluctuations would confound the intrinsic densitometric gradients and disrupt the stable thresholds essential for our tsVR model. Additionally, our tsVR model established on unenhanced CT images might have limited effectiveness in distinguishing central lung tumors from post-obstructive pneumonia due to the lack of aerated alveolar interfaces. For such complex cases not included in our current cohort, contrast-enhanced CT remains highly valuable to delineate the viable tumor from atelectatic lung tissue. Meanwhile, emerging evidence suggests that PTLC lesions are bordered by a clear stromal interface rich in tumor-infiltrating lymphocytes (TILs) and tumor-associated macrophages (TAMs), which correspond to distinct histological transitions and lower CT densities in peri-lesional regions on imaging (28, 29). In contrast, pneumonia commonly presents with perilesional inflammatory exudates or alveolar hemorrhage (30, 31). These pathological manifestations appear radiographically as areas of increased attenuation that are visually indistinguishable from adjacent pulmonary parenchyma without quantitative analysis. By objectively evaluating attenuation gradients, Koo et al. (32) also revealed a steeper decline in CT values from the lesion core to the periphery in lung cancer compared to pneumonia, aligning with the findings in our study.

This study has several limitations. First, the retrospective and fundamentally monocentric design may introduce selection bias, despite our efforts to mitigate it through consecutive enrollment and rigorous exclusion criteria. Its generalizability in real-world settings requires prospective validation through true large-scale multi-center studies with divergent imaging infrastructures. Second, our quantitative image analysis was methodologically restricted: the sampling radii were empirically defined, and we primarily focused on single dominant consolidations. Future studies should systematically investigate optimal sampling margins and the model’s efficacy in complex cases presenting with multifocal consolidations. Third, establishing direct causality between the observed densitometric gradients and microenvironmental changes requires future histopathologic correlation. Lastly, our final cohort lacked certain specific inflammatory subtypes, such as organizing pneumonia (OP). This absence, coupled with the pathophysiological diversity of benign lesions, highlights the need for further etiology-specific investigations to fully address pneumonia misclassifications.

In conclusion, the tsVR model provides an efficient and clinical applicable solution for differentiating PTLC from benign inflammatory lesions with high accuracy and robustness across diverse imaging conditions.

## Data Availability

The raw data supporting the conclusions of this article will be made available by the authors, without undue reservation.
