# Supplementary material for: Quantitative peri-lesional densitometry mapping via thin-slice volume rendering enhances differentiation of pneumonic-type lung cancer and inflammatory pneumonia
Source: Front Oncol. 2026 Apr 28;16:1620815. doi: 10.3389/fonc.2026.1620815 (PMC13160731; doi:10.3389/fonc.2026.1620815)
Supplement: Supplementary file 1 [file DataSheet1.docx]

Supplementary Material

# Performance metrics formula

Sensitivity/Recall = True Positives / (True Positives + False Negatives)

Specificity = True Negatives / (True Negatives + False Positives)

Accuracy = (True Positives + True Negatives) / (True Positives + True Negatives + False Positives + False Negatives)

Precision = True Positives / (True Positives + False Positives)

PPV = True Positives / (True Positives + False Positives)

NPV = True Negatives / (True Negatives + False Negatives)

F1-score = 2 * (Precision * Recall) / (Precision + Recall)

F2-score = 5 * (Precision * Recall) / (4 * Precision + Recall)

MCC = (True Positives * True Negatives - False Positives * False Negatives) / sqrt((True Positives + False Positives) * (True Positives + False Negatives) * (True Negatives + False Positives) * (True Negatives + False Negatives))

# Supplementary Figures and Tables

## Supplementary Figures

##
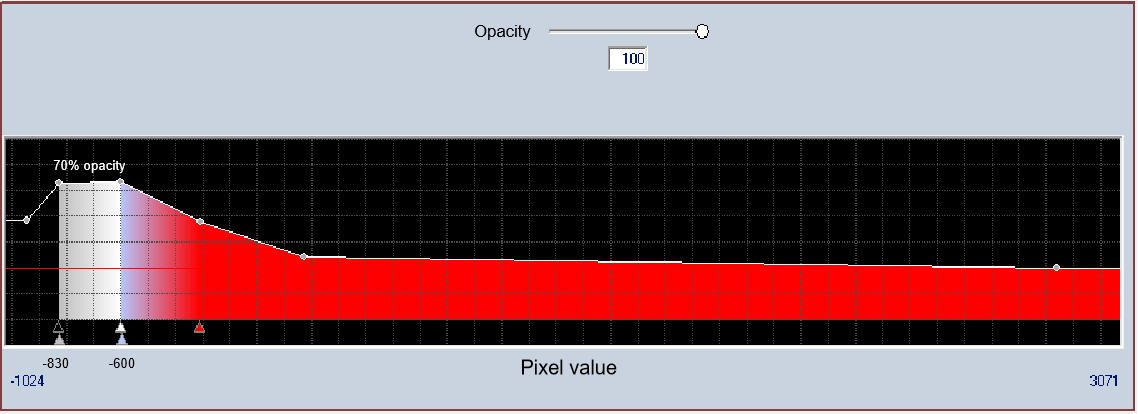


## **Supplementary Figure 1.** Opacity and psedo color curve setting of tsVR.

**2.2 Supplementary Tables**

| **Supplementary Table 1.** Patient characteristics in the training and validation cohorts | | | | | | | | | | | |  |
| --- | --- | --- | --- | --- | --- | --- | --- | --- | --- | --- | --- | --- |
|  |  | Training set | | |  | Internal validation set | | |  | External validation set | | |
| Variables |  | Pneumonia (n = 133) | PTLC (n = 135) | *P* |  | Pneumonia (n = 57) | PTLC (n = 58) | *P* |  | Pneumonia (n = 30) | PTLC (n = 31) | *P* |
| **Age, M (Q₁, Q₃)** |  | 56.00 (50.00, 66.00) | 62.00 (53.00, 68.50) | 0.002^a^ |  | 54.00 (48.00, 67.00) | 65.50 (57.25, 68.00) | 0.003^a^ |  | 64.50 (56.50, 70.50) | 66.00 (53.50, 68.00) | 0.549^a^ |
| **Gender, n(%)** |  |  |  | <.001^b^ |  |  |  | <.001^b^ |  |  |  | 0.561^b^ |
| Male |  | 99 (74.44) | 58 (42.96) |  |  | 40 (70.18) | 21 (36.21) |  |  | 25 (83.33) | 24 (77.42) |  |
| Female |  | 34 (25.56) | 77 (57.04) |  |  | 17 (29.82) | 37 (63.79) |  |  | 5 (16.67) | 7 (22.58) |  |
| **Smoking history, n(%)** |  |  |  | <.001^b^ |  |  |  | 0.155^b^ |  |  |  | 0.705^b^ |
| Smoker |  | 69 (51.88) | 99 (73.33) |  |  | 36 (63.16) | 29 (50.00) |  |  | 15 (50.00) | 17 (54.84) |  |
| Non-smoker |  | 64 (48.12) | 36 (26.67) |  |  | 21 (36.84) | 29 (50.00) |  |  | 15 (50.00) | 14 (45.16) |  |
| **Respiratory symptoms^c^, n(%)** |  |  |  | <.001^b^ |  |  |  | <.001^b^ |  |  |  | 0.034^b^ |
| With symptoms |  | 102 (76.69) | 75 (55.56) |  |  | 46 (80.70) | 29 (50.00) |  |  | 27 (90.00) | 21 (67.74) |  |
| Without symptoms |  | 31 (23.31) | 60 (44.44) |  |  | 11 (19.30) | 29 (50.00) |  |  | 3 (10.00) | 10 (32.26) |  |
| **Location, n(%)** |  |  |  | 0.878^b^ |  |  |  | <.001^b^ |  |  |  | 0.547^b^ |
| Right upper lobe |  | 34 (25.56) | 42 (31.11) |  |  | 13 (22.81) | 19 (32.76) |  |  | 8 (26.67) | 9 (29.03) |  |
| Right middle lobe |  | 14 (10.53) | 12 (8.89) |  |  | 11 (19.30) | 0 (0.00) |  |  | 4 (13.33) | 1 (3.23) |  |
| Right lower lobe |  | 29 (21.80) | 27 (20.00) |  |  | 12 (21.05) | 14 (24.14) |  |  | 7 (23.33) | 6 (19.35) |  |
| Left upper lobe |  | 38 (28.57) | 35 (25.93) |  |  | 9 (15.79) | 21 (36.21) |  |  | 5 (16.67) | 9 (29.03) |  |
| Left lower lobe |  | 18 (13.53) | 19 (14.07) |  |  | 12 (21.05) | 4 (6.90) |  |  | 6 (20.00) | 6 (19.35) |  |
| **Pathological subtype, n(%)** |  |  |  | — |  |  |  | — |  |  |  | — |
| Invasive adenocarcinoma |  | — | 113 (83.70) |  |  | — | 49 (84.48) |  |  | — | 26 (83.87) |  |
| Invasive mucinous adenocarcinoma |  | — | 5 (3.70) |  |  | — | 2 (3.45) |  |  | — | 1 (3.23) |  |
| Other (malignant) |  | — | 17 (12.59) |  |  | — | 7 (12.07) |  |  | — | 4 (12.90) |  |
| Non-specific pneumonia |  | 87 (65.41) | — |  |  | 37 (64.91) | — |  |  | 19 (63.33) | — |  |
| Tuberculosis |  | 30 (22.56) | — |  |  | 13 (22.81) | — |  |  | 7 (23.33) | — |  |
| Inflammatory pseudotumor |  | 16 (12.03) | — |  |  | 7 (12.28) | — |  |  | 4 (13.33) | — |  |

PTLC: pneumonic-type lung cancer, M: Median, Q₁: 1st Quartile, Q₃: 3st Quartile

a Mann-Whitney test

b Chi-square test

c Respiratory symptoms including fever, cough, sputum, blood in sputum, hemoptysis, and chest pain

| **Supplementary Table 2.** Influencing factors associated with diagnostic accuracy for CT | | | | | |
| --- | --- | --- | --- | --- | --- |
| **Variables** | **β** | **S.E** | **Z** | ***P*** | **OR (95%CI)** |
|  |  |  |  |  |  |
| **Diagnosis** |  |  |  |  |  |
| Pneumonia |  |  |  |  | 1.00 (Reference) |
| PTLC | 0.32 | 0.31 | 1.04 | 0.297 | 1.38 (0.76 ~ 2.51) |
| **Histological subtype** |  |  |  |  |  |
| Invasive adenocarcinomas |  |  |  |  | 1.00 (Reference) |
| Invasive mucinous adenocarcinoma | 0.45 | 0.87 | 0.52 | 0.601 | 1.58 (0.29 ~ 8.67) |
| Other | -0.17 | 0.8 | -0.22 | 0.828 | 0.84 (0.18 ~ 4.03) |
| Non-specific pneumonia | -0.39 | 0.36 | -1.09 | 0.275 | 0.68 (0.34 ~ 1.36) |
| Tuberculosis | -0.06 | 0.51 | -0.11 | 0.913 | 0.95 (0.35 ~ 2.59) |
| Inflammatory pseudotumor | -0.28 | 0.65 | -0.43 | 0.668 | 0.76 (0.21 ~ 2.71) |
| **Tumor grade** |  |  |  |  |  |
| Low |  |  |  |  | 1.00 (Reference) |
| Moderate | -1.09 | 1.15 | -0.94 | 0.345 | 0.34 (0.04 ~ 3.22) |
| High | -0.47 | 1.26 | -0.37 | 0.71 | 0.62 (0.05 ~ 7.46) |
| **Equipment** |  |  |  |  |  |
| Siemens |  |  |  |  | 1.00 (Reference) |
| GE | 0.52 | 0.32 | 1.62 | 0.105 | 1.67 (0.90 ~ 3.13) |
| Other | 0.67 | 0.65 | 1.02 | 0.307 | 1.95 (0.54 ~ 7.01) |
| **Scanning Dose** |  |  |  |  |  |
| Standard (>2 mSv) |  |  |  |  | 1.00 (Reference) |
| Low (≤2 mSv) | 0.16 | 0.38 | 0.43 | 0.667 | 1.18 (0.56 ~ 2.49) |
| **Reconstruction Kernel** |  |  |  |  |  |
| Smooth |  |  |  |  | 1.00 (Reference) |
| Medium | -0.92 | 0.33 | -2.75 | **0.006** | 0.40 (0.21 ~ 0.77) |
| Sharp | -0.9 | 0.76 | -1.19 | 0.235 | 0.41 (0.09 ~ 1.79) |
| **Reconstuction Algorithm** |  |  |  |  |  |
| IR |  |  |  |  | 1.00 (Reference) |
| FBP | -0.93 | 0.36 | -2.59 | **0.010** | 0.39 (0.19 ~ 0.80) |
| OR: Odds Ratio, CI: Confidence Interval, PTLC: pneumonic-type lung cancer, FBP: filtered back projection, IR: iterative reconstruction | | | | | |

| **Supplementary Table 3.** Influencing factors associated with diagnostic accuracy for CT | | |
| --- | --- | --- |
| **Pathological Diagnosis** | **Disease Category** | **Main Radiological Features on CT** |
| Invasive adenocarcinoma | Tumoral (PTLC) | Mixed GGO and consolidation; irregular, lobulated, or spiculated margins; pleural indentation; rigid air bronchograms; mild to moderate heterogeneous enhancement. |
| Invasive mucinous adenocarcinoma | Tumoral (PTLC) | Low-attenuation consolidation; interlobular fissure bulging; crazy-paving pattern; multifocal distribution; CT angiogram sign; mild patchy enhancement. |
| Other (e.g., squamous cell carcinoma) | Tumoral (PTLC) | Focal mass-like opacities; clear tumor-lung interface; internal necrosis or cavitation; moderate to marked enhancement. |
| Non-specific pneumonia | Non-tumoral | Patchy or lobar consolidation; ill-defined margins; halo sign; natural air bronchograms; marked homogeneous enhancement. |
| Tuberculosis | Non-tumoral | Upper lobe predilection; tree-in-bud nodularity; cavitation; calcifications; satellite lesions; peripheral rim enhancement. |
| Inflammatory pseudotumor | Non-tumoral | Solitary well-circumscribed nodule or mass; homogeneous density; marked and progressive enhancement. |
